# Founders Can Increase Determinism of Community Assembly

**DOI:** 10.1002/ece3.71428

**Published:** 2025-05-07

**Authors:** Yi‐Ting Cheng, Wei Deng, Xiao‐Yan Yang, Kun Tan, Wen Xiao

**Affiliations:** ^1^ Institute of Eastern‐Himalaya Biodiversity Research Dali University Dali Yunnan China; ^2^ Collaborative Innovation Center for Biodiversity and Conservation in the Three Parallel Rivers Region of China Dali Yunnan China; ^3^ The Provincial Innovation Team of Biodiversity Conservation and Utility of the Three Parallel Rivers Region Dali University Dali Yunnan China; ^4^ International Centre of Biodiversity and Primates Conservation Dali Yunnan China

**Keywords:** community assembly, community maintenance, dispersal, founders, microbial diversity

## Abstract

The effect of founders (the potential influence of initially colonizing species on the composition, functionality, and stability of communities) plays a crucial role in community assembly; many experimental studies on priority effects or artificially assembled species have suggested the existence of this effect, but direct experimental evidence at the community level remains limited. This study used sterilized and nonsterilized paocai soup (a traditional Chinese fermented vegetable soup) from the same source to simulate initial environments with and without founders. These were placed in beakers with varying opening sizes on an open rooftop for 15 days to explore the impact of founders on community assembly under different dispersal intensities. The 16S rRNA sequencing analysis revealed that communities with founders exhibited lower species richness (320) compared to communities without founders (645). Additionally, communities with founders showed reduced species turnover and richness variation (53.7%) compared to communities without founders (60.9%). Furthermore, the average variability degree (AVD) in communities with founders (0.446 ± 0.044) was significantly lower than in communities without founders (0.927 ± 0.466), indicating higher community stability. Finally, deterministic processes dominated communities with founders (with heterogeneous selection contributing 70%), whereas stochastic processes primarily governed communities without founders (homogeneous dispersal 10% and undominated processes 70%). These findings demonstrate that founders presence reduces dispersal impacts, decreases community diversity, enhances stability, and deterministic processes. The effect of founders fundamentally shapes the direction of community assembly. This study helps further understanding of how founders influence biodiversity maintenance and community assembly processes.

## Graphical abstract

The effect of founders exists in microbial community assembly. It significantly affects the diversity and stability of the community assembly process of the community.

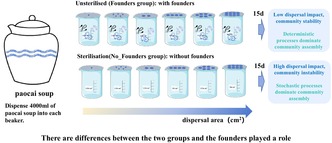

## 1 Introduction

The effect of founders refers to the potential influence of initially colonizing species on the composition, functionality, and stability of communities (Grime 1998). Notably, this concept differs from the founder effect in population genetics, which refers to the reduction in genetic variation that occurs when a new population is established by a small number of individuals from a larger population (Barton and Charlesworth 1984; Grant 2002). The founder effect also differs from the currently prominent priority effect in community assembly, which emphasizes the importance of the sequence of species immigration and involves more than just the founders (Debray et al. 2022). Exploring how the effect of founders shapes microbial communities helps to uncover the mechanisms driving the formation, maintenance, and evolution of biodiversity (Vieira et al. 2021).

The role of founders in community assembly primarily manifests through niche occupation and environmental modification, thereby influencing the dispersal, extinction, and speciation of other species (Vieira et al. 2018; Vieira et al. 2021; Debray et al. 2022). When the founders occupy ecological niches most favorable for their survival and reproduction, they create intense resource competition for incoming non‐native species. Species with overlapping niches often face significant challenges in establishing themselves, thereby reducing the success rate of non‐native species colonization (Vieira et al. 2018; Jehovah 2020). Furthermore, founders can modify the surrounding physicochemical environment through metabolic activities, which not only influence species establishment but may also drive the extinction of certain species (Miller and O'Dwyer 2024; Whitlock 1997). Over the long term, these alterations to resources and environmental conditions can shape selection pressures and influence the trajectory of new species formation (Baker 1928; Parsons 1994; Yamaguchi and Otto 2020).

Many studies have supported the existence of founder effects. The territorial behavior of animals, in which they defend spaces to monopolize resources, not only actively repels intruders of the same species but also affects the distribution and richness of other species (Betancourth‐Cundar et al. 2024; Hansen et al. 2024; Klomp 1971). Native plant species are frequently observed to dominate in competitive interactions with introduced species (Chen et al. 2024; Simberloff 2013; Vilà and Weiner 2004). Through mechanisms such as resource competition and chemical inhibition, native species exert strong constraints on competitors, thereby playing a critical role in shaping community dynamics (De Freitas and Fredrickson 1978; Pontrelli et al. 2024; Tilman 1983). For instance, certain early‐successional plants can reduce invasive species establishment by up to 97% in desert ecosystems (Abella et al. 2012). Research on microbial community assembly has revealed that, with the dispersal of non‐native species, bacteria functioning as founders can “filter” specific taxa by modifying the environment. This process leads to deterministic factors playing a dominant role in community assembly (Brislawn et al. 2019). In summary, although substantial evidence exists, it often focuses only on the mechanisms of interaction between founders and specific species, lacking communities without founders as controls. Empirical studies on the impact of founders on the fate of entire communities remain insufficient.

**FIGURE 1:**
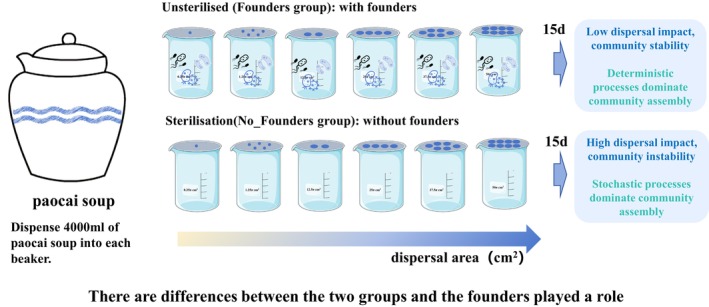
Microbial microcosmic experiment hypothesis diagram. In this study, equal amounts of homogeneous paocai soup were constructed with founders (without sterilization) and without founders (sterilization) to achieve two groups of microcosms. Each set of microcosms controls the degree of dispersal by varying the number of openings open, with the beaker gradually increasing in dispersal from left to right.

This study established two sets of open microcosm systems using paocai soup from the same source and flasks with different opening areas: communities with founders and communities without founders, to investigate the effect of founders on microbial community assembly (Figure 1). We propose that when a community with founders exists, it demonstrates stronger resistance capability, where dispersal exerts relatively minor impacts on the community. With the increase of dispersal area, community diversity typically remains low or shows no significant changes. Under these conditions, communities with founders exhibit lower diversity and higher stability compared to communities without founders, accompanied by higher determinism in the community assembly process. However, when founders are absent, communities lose their resistance capability and become significantly influenced by dispersal. As the dispersal area increases, diversity typically elevates. In this scenario, communities without founders display higher diversity and lower stability compared to communities with founders, with increased stochasticity dominating the community assembly process.

## 2 Methods

### 2.1 Experimental Design

We employed a microbial microcosm experimental framework to design and conduct this investigation.

### 2.2 Preparation of Paocai

Ten kilograms of white radish (*Raphanus sativus)*, 10 kg of cabbage ( *Brassica oleracea* ),0.6 kg of pepper ( *Capsicum frutescens* ), 0.3 kg of ginger (*Zingiber officinale)*, 0.3 kg of pepper (*Zanthoxylum bungeanum*), 0.7 kg of rock sugar, 60 kg of cold boiled water (6% salt), were mixed thoroughly and packed in paocai jars for 7 days (Deng et al. 2022; Deng, Li, et al. 2023a).

### 2.3 Handling of Paocai Soup

Paocai soup was obtained by filtering the paocai through sterile gauze. The supernatant was then collected after allowing the soup to settle for 12 h. The process of filtration, settling, and supernatant collection was repeated three times. The supernatant from all jars was combined to serve as the initial community.

### 2.4 Microbiological Sampling

Six samples were collected from the supernatant as controls (labeled “Original”). Subsequently, half of the paocai soup was autoclave‐sterilized (121°C, 30 min) to serve as the group without founders (labeled “No_Founders”), while the remaining half was designated as the group with founders (labeled “Founders”). For each treatment, the paocai soup was distributed into six 5,000 mL beakers, with 4,000 mL in each beaker. Dispersal intensity was meticulously controlled by perforating the beaker lids using two coring tools. Tool A (0.5 cm radius) created one and five openings, corresponding to areas of 0.25 and 1.25 π cm^2^, respectively. Tool B (2.5 cm radius) created 2, 4, 6, and 8 openings, with areas of 12.5, 25, 37.5, and 50 π cm^2^, respectively. All beakers were sealed with sterile newspaper and placed on an open rooftop for a 15‐day exposure period. Then samples were centrifuged at 1,200 rpm for 10 min, and the resulting pellets were stored at −80°C. The samples were then shipped on dry ice to Shenzhen Wekemo Tech Group Co. Ltd. for sequencing analysis (Deng et al. 2022; Deng, Li, et al. 2023a).

### 2.5 Analysis of Microbial Sequencing Data

#### 2.5.1 DNA Extraction and PCR Amplification

Sequencing services were provided by Wekemo Tech Group Co. Ltd.

Shenzhen, China, using the E.Z.N.A. Soil kit (Omega Bio‐tek, Norcross, GA, USA) to extract microbial DNA from the samples, the NanoDrop 2000 kit to check DNA concentration and purity, and 1% agarose gel electrophoresis to check DNA extraction quality. Bacteria were PCR amplified using 338 F (5′‐ACTCCTACGGGAGGCAGCAG‐3′) and 806 R (5′‐GGACTACHVGGGTWTCTAAT‐3′) primers targeting the V3–V4 variable region of the 16S ribosomal RNA (rRNA) gene (DeSantis et al. 2006; Jovel et al. 2016).

#### 2.5.2 Amplification Systems

PCR reaction system: 20 μL total volume, containing 4 μL of 5× FastPfu buffer, 2 μL of 2.5 mM dNTPs, 0.8 μL of forward primer (5 μM), 0.8 μL of reverse primer (5 μM), 0.4 μL of FastPfu polymerase, 0.2 μL of BSA, and 10 ng of template DNA, made up to 20 μL with ddH2O. The PCR program was as follows: initial denaturation at 95°C for 3 min, 27 cycles (denaturation at 95°C for 30 s, annealing at 55°C for 30 s, and extension at 72°C for 45 s), and final extension at 72°C for 10 min.

#### 2.5.3 Illumina NovaSeq Sequencing

PCR products were extracted from 2% agarose gels and further purified using the AxyPrep DNA Gel Extraction Kit from Axygen Biosciences, Union City, CA, USA. The purified DNA was eluted in Tris–HCl buffer and then detected via 2% agarose gel electrophoresis. Libraries were constructed utilizing the Illumina TruSeq DNA PCR‐Free Library Preparation Kit, also from Axygen Biosciences, Union City, CA, USA. After quantification with Quantiflur‐ST, the qualified libraries were sequenced on the NovaSeq 6000 platform using the PE250 sequencing protocol (Bokulich et al. 2018).

#### 2.5.4 Data Processing

The initial step involved importing raw FASTQ files into the QIIME2 platform. Within this environment, sequences from each sample were meticulously processed, including identification, stringent quality filtering, precise trimming, effective denoising, and seamless merging, all facilitated by the QIIME2 dada2 plugin. This comprehensive approach enabled the generation of feature tables comprising amplicon sequence variants (ASVs), which are the variants of the amplified subsequences (Callahan et al. 2016). Then, the QIIME2 feature‐classifier plugin was employed to match the representative sequences of ASVs against the pretrained version 13_8 of the GREENGENES database, which boasts a 99% similarity threshold. This database was tailored to the V3–V4 region, aligned with the 338F/806R primer pairs, and a 70% matching threshold was applied to secure a robust taxonomic information table for species identification (Bokulich et al. 2018). In a subsequent step, the QIIME2 feature‐table plugin was utilized to meticulously eliminate all contaminating sequences originating from mitochondria and chloroplasts. Additionally, rare ASVs, which constituted less than 0.001% of the total sequences, were excluded to maintain the integrity and reliability of the dataset (Callahan et al. 2016; Marizzoni et al. 2020).

#### 2.5.5 Data Analysis

All results were done using R_4.4.1. The ggplot2 (https://ggplot2.tidyverse.org), vegan (https://CRAN.R‐project.org/package=vegan), and VennDiagram (https://CRAN.R‐project.org/package=VennDiagram) packages were utilized for analysis. The analysis involved the use of dominant genus bar stacking plots and community composition Venn diagrams to show the type and amount of variation in species composition, and PCoA (principal coordinates analysis) results were analyzed using the ade4 package to illustrate the differences between samples further (Bougeard and Dray 2018). To analyze the sources of differences among samples, the ade4 package was used to perform β‐diversity partitioning to evaluate the relative contributions of species replacement or richness difference to β‐diversity (Baselga and Orme 2012). Community stability was calculated using the average variability degree (AVD) index:

$$\mathrm{AVD}=\frac{\sum_{i=1}^{n}\frac{\left|x_{i}-\left.\bar{x_{i}}\right|\right.}{\delta_{i}}}{k\times n}$$

k is the number of samples in the group and n is the number of ASVs. $x_{i}$ is the abundance of ${\mathrm{ASV}}_{i}$ in each sample, and $\bar{x_{i}}$ and $\delta_{i}$ are the mean and standard deviation of the abundance of ${\mathrm{ASV}}_{i}$ in all samples, respectively (Xun et al. 2021). The robustness of microbial ecological networks was defined as the proportion of species remaining in the network following random or targeted removal of nodes. Networks were constructed, and a linear relationship between the removal of nodes and the proportion of remaining species was plotted. The slope of the fitted straight line was then calculated to indicate community stability. This was achieved using the ggplot2, dplyr, and the microeco packages. The four basic ecological processes of selection, dispersal, speciation, and undominated processes were decomposed and quantified using the Stegen et al. (2013) community assembly analysis framework provided in the iCAMP package. Specific partitioning principles were determined by pairwise comparisons of βNTI (β‐nearest‐taxon index) based on the Raup–Crick metric of Bray–Curtis distance (RC). When |βNTI| < 2, RC < −0.95 indicates homogeneous dispersal; |βNTI| < 2 and RC > 0.95 represent the relative influence of dispersal limitations. |RC| < 0.95 represents an undominated fraction (not dominated by a single process) (Stegen et al. 2013).

## 3 Results

Anomalous microcosmic sequencing results for 0.25 π cm^2^, suspected to be sequencing contamination, were excluded from subsequent analyses in order to ensure the reliability of the data studied. High‐throughput sequencing yielded a total of 115,184 high‐quality sequences containing 790 ASVs. There were 215 ASVs found in the original communities, 320 ASVs in the community with founders, and 645 ASVs in the community without founders. The sequencing depth of 50,000 sequences was used.

### 3.1 Significant Differences Exist in Species Composition Between Communities With and Without Founders

**FIGURE 2:**
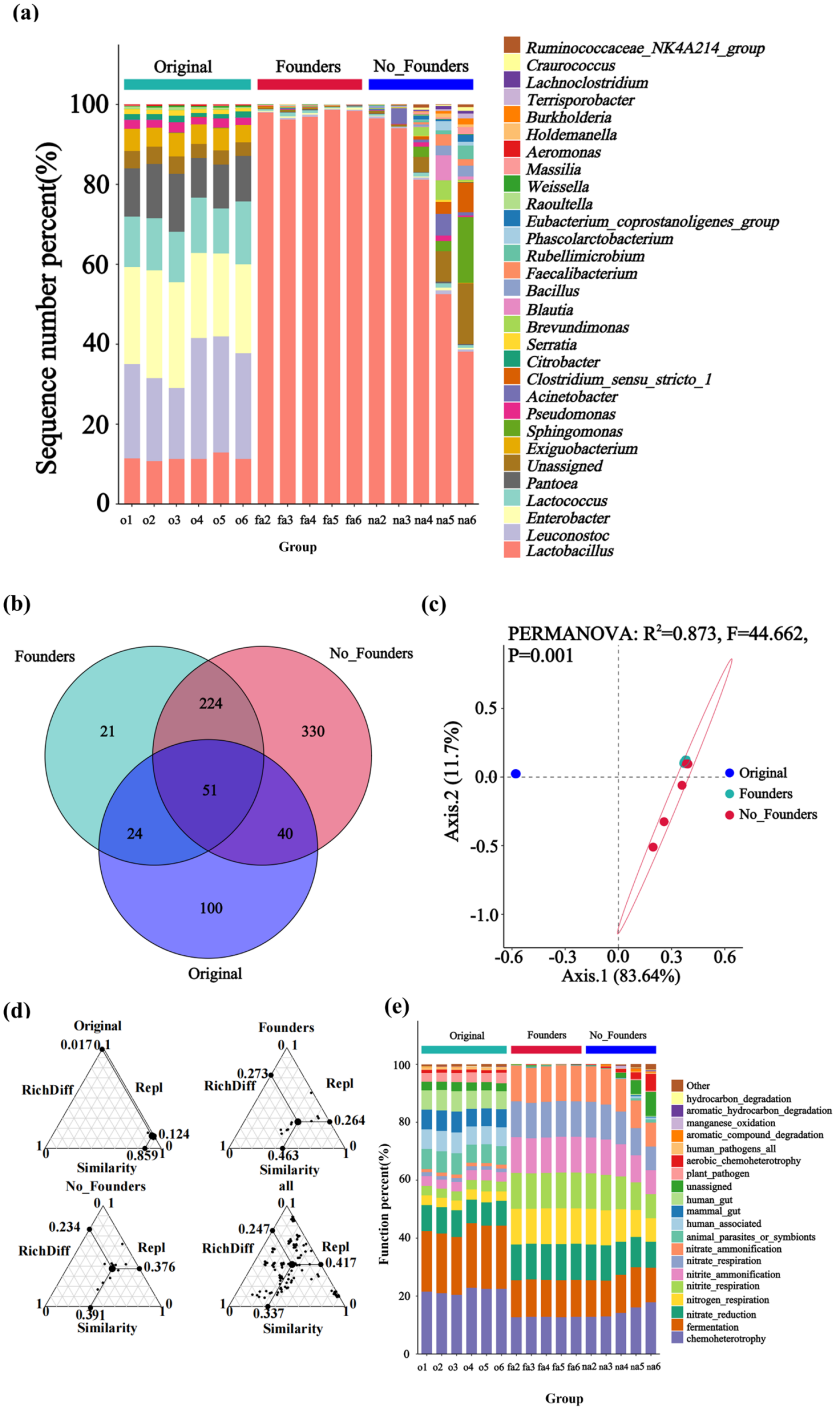
Microbial community characteristics: (a) Genus‐level composition (stacked bars) showing the relative abundance of dominant taxa, with color codes representing specific genera. This study defined genera with a cumulative relative abundance exceeding 1% across all samples as dominant genera. (b) Venn diagram illustrating ASV distribution among groups (overlaps = shared ASVs; nonoverlapping regions = unique ASVs). (c) PCoA ordination demonstrating β‐diversity patterns (point proximity reflects community similarity). (d) β‐diversity partitioning: RichDiff (richness difference), Repl (species replacement), and similarity components (percentage values indicate contribution weights). (e) Functional potential analysis (stacked bars) displaying predominant metabolic pathways (color codes = functional categories).

Here the genera with an average relative abundance greater than 1.0% within the same treatment group were defined as dominant genera. In the original community, a total of nine dominant genera were identified: *Unclassified_Enterobacteriaceae* (relative abundance is 28.2%), *Leuconostoc* (22.3%), *Lactococcus* (11.7%), *Lactobacillus* (10.4%), *Enterobacter* (6.4%), *Pantoea* (6.4%), *Rahnella* (4.7%), *Exiguobacterium* (4.7%), and *Citrobacter* (1.1%). In the community with founders, only *Lactobacillus* was identified as a dominant genus, with a relative abundance of 95.7%. In contrast, the community without founders contained seven dominant genera: *Lactobacillus* (66.3%), *Sphingomonas* (3.4%), *Acinetobacter* (2.2%), *Clostridium_sensu_stricto_1* (2.0%), *Brochothrix* (1.3%), *Blautia* (1.2%), and *Unclassified_Caulobacteraceae* (1.2%). Among these, *Lactobacillus* was the only genus shared as a dominant genus in the three groups. In the samples without founders, the relative abundance of *Lactobacillus* decreased while the number of dominant genera increased with the dispersal area (Figure 2a). The most unique species were found in the communities without founders (330), followed by the original communities (100), and the fewest in communities with founders (21). There were 75 species shared by original communities and communities with founders, 275 species shared by original paocai soup and communities without founders, and 91 species shared by paocai soup with and without founders (Figure 2b). Original communities were primarily defined by functions such as chemoheterotrophy, fermentation, and nitrate reduction, along with mammalian or human gut‐associated environments. Communities with founders developed enhanced nitrogen cycling capacities, including nitrogen respiration and nitrite ammonification. In contrast, communities without founders exhibited a high proportion of unassigned functions and aerobic chemoheterotrophy. Notably, communities without founders showed dispersal intensity‐dependent functional expansion, whereas communities with founders maintained relatively stable functional profiles across dispersal gradients (Figure 2e).

Significant differences in bacterial communities were observed among original communities, communities with founders, and communities without founders (*R* ^*2*^ = 0.873, *F* = 44.662, *p* = 0.001). Both communities with founders and original communities showed low within‐group variation (Bray–Curtis distances: 0.103 ± 0.025 vs. 0.039 ± 0.014, average ± standard deviation), whereas communities without founders exhibited greater variability (0.446 ± 0.191) (Figure 2c). The overall β‐diversity was 66.3%, while the original communities, communities with founders, and communities without founders were 14.1%, 53.7%, and 60.9%, respectively. Richness difference and species replacement contributed 1.7% and 12.4% to β‐diversity in original communities, 27.3% and 26.4% in communities with founders, and 23.4% and 37.6% in communities without founders (Figure 2d).

### 3.2 Founders Maintain Stability of Communities

**FIGURE 3:**
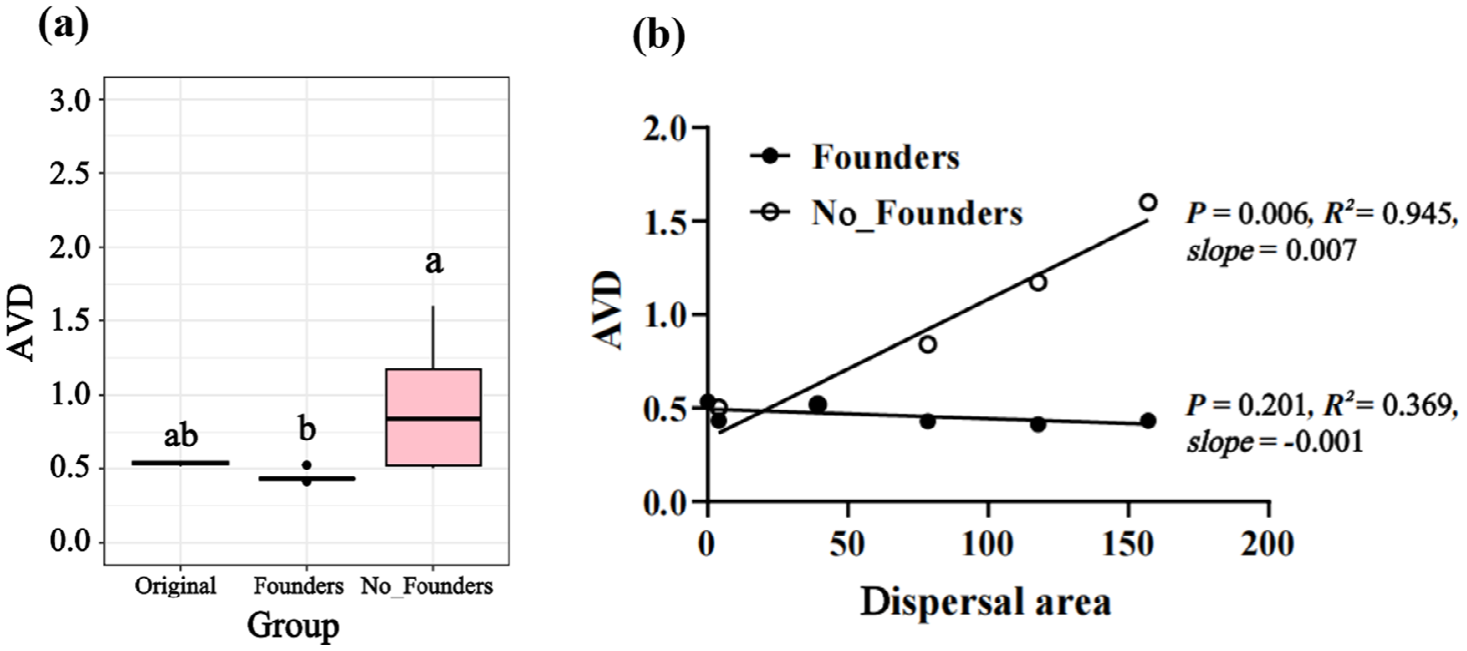
Changes in communities stability with increasing dispersal area: (a) Comparing the stability of the original communities, the communities with founders, and the communities without founders after 15 days of free assembly; (b) changing patterns of community stability with increasing dispersal area.

**FIGURE 4:**
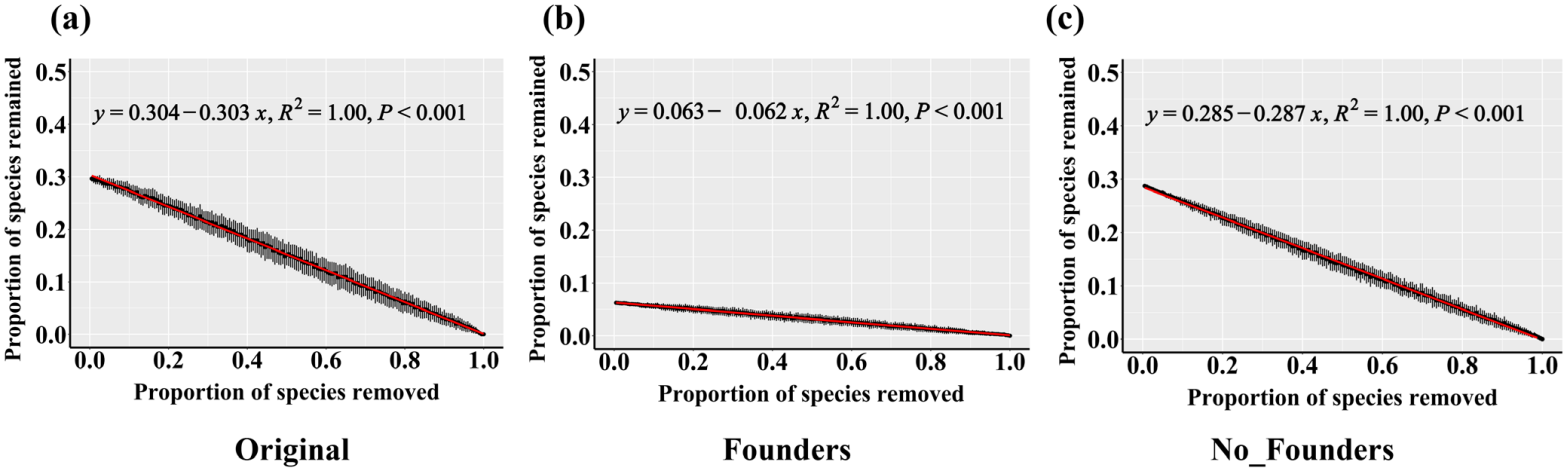
Robustness analysis of the microbial cooccurrence network: (a) Original communities; (b) communities with founders; (c) communities without founders. The horizontal coordinate is the proportion of randomly removed species, and the vertical coordinate is the proportion of remaining species.

The AVD indices among the original communities (0.535 ± 0.013), communities with founders (0.446 ± 0.044), and communities without founders (0.927 ± 0.466) were significantly different (df = 2, *F* = 4.932, *p* = 0.026). There were no significant correlations between dispersal area and AVD (*R* ^2^ = 0.369, *slope* = −0.001; *p* = 0.201) in the communities with founders. In contrast, communities without founders AVD indices increased with dispersal area (*R* ^2^ = 0.945, *slope* = 0.007; *p* = 0.006), reflecting reduced stability (Figure 3a,b). Network robustness analysis revealed that original communities contained the most network‐forming species, followed by communities without founders, with communities with founders having the fewest. The slopes of remaining species decline during node removal were 0.303 (original communities), 0.287 (communities without founders), and 0.062 (communities with founders), demonstrating the slowest network collapse rate in communities with founders (Figure 4).

### 3.3 Changes in Community Diversity With Increasing Dispersal Area

**FIGURE 5:**
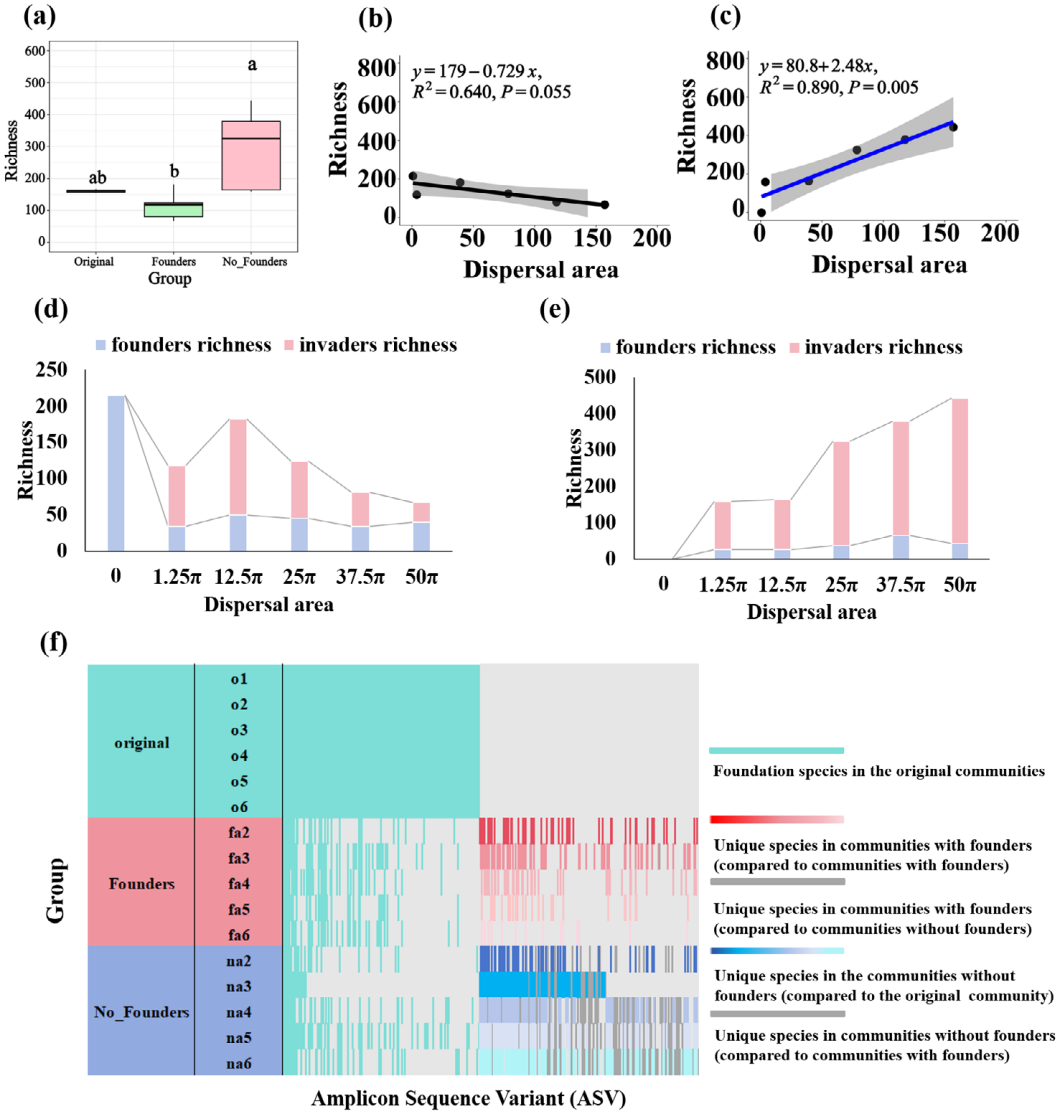
Changes in community diversity with increasing dispersal area: (a) Comparison of species richness among original communities, communities with and without founders after 15 days of free assembly. (b) In communities with founders, species richness showed no significant change with increasing dispersal area. (c) In communities without founders, species richness exhibited a significant increase with expanding dispersal area. (d) In communities with founders, changing trends of founder and invader richness with dispersal area expansion. (e) In communities without founders, changing trends of founder and invader richness with dispersal area expansion. (f) ASV dissimilarity comparison among original communities, communities with founders, and communities without founders. The “0” in the *x*‐axis labels denotes the original communities.

Overall, communities without founders exhibited substantially higher species richness than those with founders, while communities with founders showed closer resemblance to the original communities (Figure 5a). In communities with founders, species richness exhibited no significant change with increasing dispersal area (*R* ^*2*^ = 0.640, *slope* = −0.729; *p* = 0.055) (Figure 5b). Conversely, communities without founders demonstrated significant increases in species richness with expanding dispersal area (*R* ^*2*^ = 0.890, *slope* = 2.48; *p* = 0.005) (Figure 5c). The microorganisms in the community were distinguished into founders (taxa that were present in the original communities) and invaders (taxa that were not present in the original communities). In the communities with founders, the richness of founders first decreased and then remained at a steady level as the dispersal area increased, while the richness of invaders first increased and then decreased (Figure 5d,f). In the communities without founders, the richness of founders increased slightly with increasing dispersal area and then remained at a flat level; the richness of invaders continued to increase (Figure 5e,f).

### 3.4 Founders Increase Deterministic Processes for Microbial Community Assembly

**FIGURE 6:**
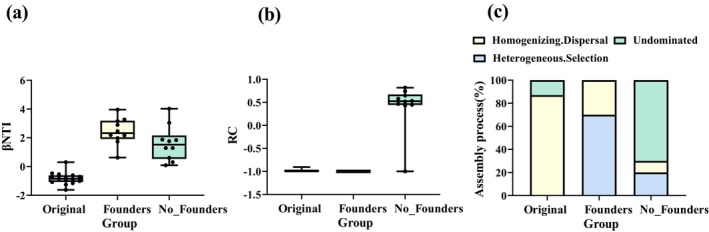
Analysis of microbial community assembly process: (a) βNTI values; (b) RC values; (c) proportional contributions of assembly process.

The original communities showed βNTI and RC values of −0.821 ± 0.431 and −0.984 ± 0.029, respectively. For communities with founders, the values were 2.444 ± 0.935 (βNTI) and −1 (RC), while communities without founders exhibited 1.611 ± 1.214 (βNTI) and 0.417 ± 0.513 (RC). Regarding βNTI index, both communities with and without founders demonstrated βNTI values > 0, significantly higher than the original communities (βNTI < 0). Notably, communities with founders showed the highest βNTI values (> 2). The RC index revealed similar values between communities with founders and original communities, whereas communities without founders displayed the highest RC value (Figure 6a,b). According to Stegen et al.'s (2013) framework for community assembly: (1) original communities were primarily governed by homogeneous dispersal (87%) with undominated processes (13%) (stochasticity dominated). (2) Communities with founders showed assembly mechanisms dominated by heterogeneous selection (70%) and homogeneous dispersal (30%) (deterministic processes prevailing). (3) Communities without founders comprised undominated processes (70%), heterogeneous selection (20%), and homogeneous dispersal (10%) (Figure 6c).

## 4 Discussion

Our study found that after 15 days of dispersal and assembly, microbial communities with founders exhibited significant differences in diversity, taxa, and functional composition compared to the initial communities without founders. Additionally, communities with founders were more stable and had a higher proportion of deterministic processes dominating their assembly. These findings support the effect of founders.

Founders were likely to significantly reduce the proportion of airborne microbial colonization. The number of unique ASVs in communities without founders was as high as 15.7 times that in communities with founders (330 vs. 21). Comparing this with the study conducted by our team using filter paper to collect airborne microbial data in the same area (Deng, Yu, et al. 2023b), it was found that only 15.3% of ASVs successfully colonized in communities with founders, whereas this rate was 22.4% in communities without founders. At the dominant genus level, this resistance was particularly significant for the genera *Brevundimonas* and *Burkholderia*, which could only be found in communities without founders. Moreover, founders can also promote the proliferation of specific taxa. For example, in communities with founders, the average relative abundance of *Lactobacillus* was 96.0%, which was 43.6% higher compared to communities without founders. The presence of founders may restrict the dispersal process by occupying ecological niches and altering the environmental regulation of extinction, thereby determining which species can successfully colonize the community. Notably, this phenomenon also influences the functional diversity of the community, as communities without founders encompass a broader range of functional categories. Competition for ecological niches between invaders and native species is a significant characteristic of the dynamic changes in ecosystems (Halassy et al. 2023; Shan and Hou 2023). The dominant position of established species within ecological niches strongly restricts the colonization of newcomers, especially in communities with founders. Conversely, in the absence of founders, due to the lack of such interactions, a broader array of species can establish and thrive within the community, shaping higher α‐ and β‐diversity (Fukami and Nakajima 2013; McPeek 1996).

The results suggest that founders can enhance community stability: by assessing community variability, we found that communities with founders exhibited a 51.9% reduction in AVD values. This suggests that communities with founders possess resistance to external disturbances, maintaining relatively stable species composition when facing dispersal and environmental changes. Co‐occurrence network robustness analysis provided consistent evidence: the slope of remaining species decline during node removal was lowest in communities with founders, indicating the slowest network collapse rate. We propose that this pattern arises from founders' ability to influence invasion success and subsequent community dynamics (Vieira et al. 2021). When founders dominate communities, they may preempt ecological niches and modify environments, limiting resource availability and space for invaders, and thereby maintaining stable species composition (Vieira et al. 2018; Vieira et al. 2021). We also found that founders can enhance the determinism and predictability of community assembly. Studies have shown that the assembly of communities without founders is primarily driven by stochastic processes, with undominated processes and homogeneous dispersal accounting for 70% and 10%, respectively, while heterogeneous selection only accounts for 20%. This is in stark contrast to communities with founders, whose assembly is dominated by deterministic processes, with homogeneous dispersal increasing to 30% and heterogeneous selection significantly rising to 70%. The presence of founders may lead to niche compression or specialization in the environment, resulting in specific selection pressures. Such pressures may allow the survival of only a subset of species, thereby reducing the uncertainty caused by random dispersal. Existing theories and empirical studies suggest that different founder combinations can have significant effects on community assembly processes (Vieira et al. 2018; Hu et al. 2025; Huet et al. 2023).

By establishing identical initial environments for both community types and accounting for the dual influences of environmental conditions and stochastic dispersal, we uncovered fundamentally distinct community assembly outcomes between systems with and without founders under identical dispersal conditions. Our findings provide strong support for the hypothesis that the presence of founders plays a pivotal role in determining the trajectory of community succession. Communities with founders exhibited reduced diversity but enhanced stability and predictability. Founders effectively diminish the influence of dispersal intensity on community assembly, with communities containing founders displaying lower sensitivity to dispersal intensity in terms of both community composition and structural stability (Pontrelli et al. 2024; Suzuki and Economo 2024). Nevertheless, factors such as the diversity and size of the founder species pool, as well as variations in initial environmental conditions, may significantly alter community assembly dynamics (Hu et al. 2025; Huet et al. 2023). Beyond preempting ecological niches to suppress invasions, founders may influence community trajectories through environmental modifications (Lau 2008; Vieira et al. 2018; Vieira et al. 2021). However, due to the absence of specific data on environmental variables and bacterial interactions in the present study, the underlying mechanisms remain unresolved. Future research should prioritize investigations across diverse environmental gradients, varying founder compositions, and differing dispersal intensities. Long‐term monitoring of species composition, environmental parameters, and community functions is also critical to comprehensively evaluate the universality of the founder effect across a range of ecological contexts.

## 5 Conclusion

This study supports the existence of the effect of founders in microbial community assembly. Communities with founders exhibited lower diversity, higher stability, and insensitivity to dispersal intensity. In contrast, communities without founders showed higher species richness and functional expansion with increasing dispersal areas but lower stability. Communities with founders were dominated by deterministic processes, while communities without founders were primarily influenced by stochastic processes. Functionally, founders enhanced nitrogen cycling and maintained stable functional profiles across dispersal gradients, whereas communities without founders exhibited higher proportions of unassigned functions and functional expansion. In conclusion, founders play a vital role in stabilizing community structure, promoting deterministic processes, and enhancing functional robustness. Future studies should investigate the mechanisms of founder effects under varying conditions.

## Author Contributions

**Yi‐Ting Cheng:** conceptualization (equal), data curation (equal), formal analysis (equal), investigation (equal), methodology (lead), visualization (equal), writing – original draft (lead), writing – review and editing (equal). **Wei Deng:** formal analysis (equal), investigation (equal), visualization (equal), writing – review and editing (equal). **Xiao‐Yan Yang:** funding acquisition (equal), investigation (equal), supervision (equal), writing – review and editing (equal). **Kun Tan:** conceptualization (equal), funding acquisition (equal), investigation (equal), supervision (equal), writing – review and editing (equal). **Wen Xiao:** conceptualization (equal), funding acquisition (equal), investigation (equal), project administration (lead), supervision (equal), writing – review and editing (equal).

## Conflicts of Interest

The authors declare no conflicts of interest.

## Data Availability

Paocai microbiome 16S amplicon sequencing data are released (GSA: CRA008326). Please access it from the following link: https://bigd.big.ac.cn/gsa/browse/CRA008326.
